# Outsourcing cognition: the psychological costs of AI-era convenience

**DOI:** 10.3389/fpsyg.2025.1645237

**Published:** 2025-12-05

**Authors:** Binny Jose, Deepak Joseph, Visakh Mohan, Elizabeth Alexander, Subi K. Varghese, Abhijith Roy

**Affiliations:** 1 Department of Health and Wellness, Marian College Kuttikkanam Autonomous, Kuttikkanam, India; 2 St. Berchmans College, Kottayam, India; 3 School of Social Work, Marian College Kuttikkanam Autonomous, Kuttikkanam, India; 4 PG Department of Social Work, Kuriakose Elias College, Kottayam, India; 5 Kristu Jyoti College of Management and Technology, Kottayam, India; 6 Sienna Senior Living, Toronto, ON, Canada

**Keywords:** cognitive off-loading, memory, attention, metacognition, identity domains, cognitive autonomy, assistive, substitutive

## Introduction

While AI technology is increasingly integrated into all aspects of thinking, learning, and decision-making, the technologies supporting those processes are going from simply enhancing our cognitive abilities to changing them. Such technologies as generative writing, searching for information on the Internet, voice recognition systems, and adaptive tutoring systems; all have made possible unprecedented efficiency in doing so. However, the increasing use of such technology has raised questions about the relationship between the field of psychology and the impact of such technology. In particular, if the need to remember, to think about what we have done, or to engage in higher order thought is lessened by the use of AI technology, then are we creating a culture of convenience that reduces our ability to be resilient cognitively?

This article examines the developing trend of “cognitive off-loading” through the use of AI technology, and the implications for the memory, attention, metacognition, and identity domains that collectively define cognitive autonomy. The approach of this article is integration; that combines current findings in cognitive offloading with existing psychological theories related to memory, metacognition and motivation. A systematic taxonomy of different forms of offloading is analyzed, distinguishing between assistive, substitutive, and disruptive forms of delegating tasks, and examining the psychological costs associated with each. This paper, as an *Opinion Article* aims to synthesize emerging psychological frameworks and empirical findings on cognitive offloading to propose an integrative conceptual taxonomy.

## The psychology of cognitive offloading

Cognitive offloading refers to using external tools to ease internal cognitive demands. Classic research such as the “Google Effect” demonstrated that when people expect information to be easily accessible online, they are less likely to remember it themselves (Sparrow et al., 2011). Later replication studies revealed that people tend to remember where information is stored rather than what it is (Camerer et al., 2016). Building on these findings, Risko and Gilbert (2016) argued that digital technologies not only alter memory but also redistribute attention and mental effort. More recent work extends this to AI tools, showing shifts in memory encoding, problem-solving strategies, and even goal formation (Grinschgl and Neubauer, 2022).

The research shows there is a spectrum of how much an external aid will affect someone's cognition (assistive, substitutive, and sometimes disruptive). Assistive offloading happens when technology helps cognition but does not interfere with cognition (example would be reminders apps or digital sticky note reminders for memory cues).

Substitutive offloading occurs when technology replaces cognition; examples include auto-suggest or predictive search which reduces cognitive processing, thus also reducing the amount of cognitive energy applied to the original source material. Disruptive offloading is the same as substitutive offloading, however, it encourages a paradigm of passive interaction where one loses their ability to mentally control what they think about and the ability to reflect upon what they think about through automated results.

Eventually regular reliance on AI for basic cognitive tasks, over time, can cause the relationship between external help and internal mental control to become unbalanced. In turn, the type of thinking people develop can become optimized for speed rather than comprehension. Understanding the differences in levels of influence for technology is important in determining when a person relies on technology to assist their cognition vs. when technology substitutes for cognition.

## From external aids to cognitive substitution

Historically, cognition was supplemented by tools like notebooks or calculators. These served as scaffolds—tools external to cognition but that facilitated greater internal processing. Contemporary AI tools, however, circumvent as opposed to augment internal cognition. Models like ChatGPT aid in essay writing, choice making, and problem solving and operate as cognitive surrogates rather than as scaffolds providing ready-made solutions.

Unlike the past generation of instruments that required the active participation of the user, and a high level of critical and reflective thought to produce original output; generative AI systems are able to automatically generate output with minimal need for ideation or reflection on part of the user. An example is the difference between an outline software that assists the user in creating ideas, and an AI tool capable of producing a full essay without the need for any thought at all. Users who start with AI generated work may avoid some of the important cognitive processes that occur when writing, such as synthesizing new thoughts, or revising existing ones (Huff and Ulakçi, 2024). These lost cognitive processes can also cause the loss of neural pathways associated with higher order thought over time, particularly if the user uses the AI tools frequently and with no discretion.

## Implications for memory and learning

The use of cognitive offloading is detrimental to the ability to remember information long-term (Lu et al., 2020), as well as the willingness to become engaged in content (Kelly and Risko, 2022) because individuals will have an expectation that the information is being held outside of themselves and therefore will be less likely to commit it to memory. A relationship exists between excessive use of artificially generated answers in educational environments, and surface-level learning, poor self-evaluation, and shallow conceptual understanding (Chiu, 2024).

Furthermore, the retrieval process—a critical component of durable learning (Karpicke and Blunt, 2011)—will most certainly be avoided if a learner relies on artificial intelligence-based explanations for their work instead of generating the knowledge through independent means. AI systems that are designed to emphasize correct answers at the expense of cognitive expenditure may also facilitate this trend, and encourage learners to move away from the pursuit of expertise toward the production of answers. Together, these patterns of digital memory use reveal that offloading rarely affects recall in isolation; it reshapes how attention is allocated to information in the first place.

## The role of metacognition and agency

Cognitive offloading is successful based on metacognitive regulation—The ability to observe and control when and how you are going to offload your cognitive workload. The AI system creates an illusion of competence and contributes to a user overestimating their awareness of what was generated and/or reviewed by the AI system. As a result, introspection accuracy and user control decrease (Hoch et al., 2023). Both decreased introspection accuracy and user control impede adolescents and young adults whose executive functioning has not yet been developed (Iley and Medimorec, 2024; Sun, 2024).

The results of research conducted using Judgment Of Learning (JOL) tests reveal that users typically overestimate the amount of knowledge they possess regarding the output produced by an AI (Hu et al., 2019). This miscalibration may reduce the need for feedback loop revisions, and therefore limit the depth of learning. In addition, as AI continues to eliminate ambiguity, the users will become increasingly intolerant of ambiguity—reducing the developmental potential of epistemological resilience. As metacognitive regulation becomes increasingly externalized, the broader question arises of how such dependency influences sustained attention and the capacity for self-directed learning.

## Cognitive effort and digital habits: the changing threshold of attention

As people routinely use AI tools for their everyday needs, their mental expectations about how much effort is required to accomplish something are altered. As an example, when there is an abundance of accessible information, what constitutes “effort” becomes elevated. In addition, as AI tools become easier to use and provide instant gratification, many will choose to avoid those activities that appear to require higher levels of cognitive engagement. Research has demonstrated that frequent exposure to digital multitasking and continuous availability of information negatively affect sustained attention and cognitive flexibility. Multitasking in a digital environment creates an unstable state of affairs among the various attentional networks. This instability makes maintaining focus and adapting to changing cognitive requirements more difficult (Lee and Schumacher, 2024).

On the other hand, while some individuals may rapidly respond to a multitude of tasks simultaneously, research indicates these same individuals make more mistakes and demonstrate poorer attentional control (Figueroa et al., 2014). Developmentally-based research also supports the idea that sustained attention is fundamental to developing cognitive flexibility, therefore early or prolonged distractions can have negative long-term effects (Benitez et al., 2017). The reward systems used by AI tools (i.e., rapid feedback, little resistance, and expected outcomes) create an inclination toward ease vs. depth. When ease is attained with less effort (e.g., difficulty in formulating a question), one may discontinue engaging in high-level thinking. These patterns of attentional adaptation under AI influence set the stage for understanding how developing minds, particularly adolescents, navigate delegated cognition.

## Adolescence and the long-term risks of delegated thinking

Cognitive offloading impacts all age ranges; however, it is possible that cognitive offloading has a greater impact on individuals at developmental stages where their Executive Functions are continuing to develop (Meunier-Duperray et al., 2025). Therefore, while there are many different types of developmental stages that exist in terms of how the brain develops and matures, adolescence represents a unique example of a developmental stage—where both the substitutes and disruptions of offloading can have significant and lasting impacts on the individual's cognitive abilities and identity formation (Bai et al., 2023).

During adolescence, many adolescents use AI technology for both academic and recreational uses; therefore, they are often among the first generation of “early adopters” of delegated cognition. The core executive functions (i.e., planning, impulse control, and self-regulation), which are the foundation of many of the cognitive processes necessary for effective learning and decision-making, are still developing throughout adolescence (Iley and Medimorec, 2024). Therefore, relying consistently on AI to either generate, organize, or evaluate information may interfere with the normal maturation process of these skills. This type of phenomenon represents what we have termed a developmental displacement effect, which refers to a situation where one of the fundamental cognitive processes (in this case, internal processing) is externally displaced prior to achieving internal mastery (Sun, 2024).

There are now empirical studies that have demonstrated this phenomenon. For example, a study by Sun et al. (2024) found that heavy AI use in school-related tasks resulted in lower levels of self-monitoring and poorer metacognitive accuracy over time. The results of this study represent one of the examples of the Disruptive Offloading level of our taxonomy, which involves the substitution of automation for internal regulation and reflection. In addition to the negative impact that premature delegation can have on short-term learning, it also can result in delayed development of independent cognitive agency, which is an important aspect of both autonomy and motivation.

Therefore, framing adolescence as a specific example to illustrate our points does not limit, but instead supports our position that the psychological consequences of cognitive offloading are developmentally graded, and that understanding these gradients is essential to creating educational and technological systems that foster—and do not impede—mental independence and self-reliance. These developmental differences highlight why a structured taxonomy of cognitive offloading is necessary—to clarify when delegation supports growth and when it begins to undermine cognitive autonomy.

## A taxonomy of cognitive offloading

Cognitive offloading is not a homogeneous process, and its psychological effects depend on the number of internal operations replaced and which cognitive operations are operant. Drawing on previous literature, we present a taxonomy which separates three kinds of offloading—assistive, substitutive, and disruptive—and relates each type to the different basic domains of cognition: memory, metacognition, attention, and learning autonomy (Table 1).

**Table 1:** Different levels of offloading redistribute cognitive labor.

**Type/level of offloading**	**Memory**	**Metacognition**	**Attention**	**Learning autonomy**
Assistive	External aids cue or reinforce recall (e.g., reminders, digital notes; Soares et al., 2023)	User retains monitoring and regulation of tool use (Jose et al., 2025)	Supports focus by reducing overload without fragmenting attention (Bai et al., 2023)	Strengthens autonomy through scaffolding; tool remains under conscious control
Substitutive	Encoding and retrieval decline when AI provides ready answers (Risko and Gilbert, 2016)	Creates an *illusion of competence*; users overestimate understanding (Tezer, 2025)	Promotes shallow engagement or multitasking (Jose et al., 2025)	Reduces agency as effort shifts to the system rather than the self (Bai et al., 2023)
Disruptive	Long-term recall and reconstruction skills erode through chronic reliance	Self-monitoring diminishes; reflection loops collapse (Jose et al., 2025)	Attention becomes externally driven and reward-seeking (Murtaza et al., 2022)	Undermines self-regulation, fostering dependency and passivity (Murtaza et al., 2022)

This structure makes explicit how different levels of offloading redistribute cognitive labor.

The three types of assistive off-loading that occur are in-line with the principles of scaffolded learning as they provide an increase in task performance with a continued level of internal engagement by the learner.

Substitute off-loading has identified the presence of measurable cognitive cost associated with using off-loads. This finding is consistent with research concerning the Google Effect and the reduction in semantic effort (Kelly and Risko, 2022; Sparrow et al., 2011).

Disruptive off-loading is a form of off-loading which results in a qualitatively different type of impact than either of the two other forms of off-loading. Specifically, Disruptive off-loading results in an undermining of metacognitive accuracy and sustained attention as well as creating a higher risk of cognitive disengagement among developing minds (Sun et al., 2024).

Using this taxonomy allows researchers to develop a theoretical framework to examine how technology is used to delegate tasks, as well as when the use of technology for delegating tasks is likely to be beneficial or detrimental to learners' cognitive development. In addition, future empirical studies will be able to operationalize the levels of off-loading, to help define at what point in time each of the three levels of off-loading result in an increase in task completion, but also an increase in cognitive costs, and to ultimately guide educators in designing responsible and effective technologies for supporting learners.

**Figure 1:**
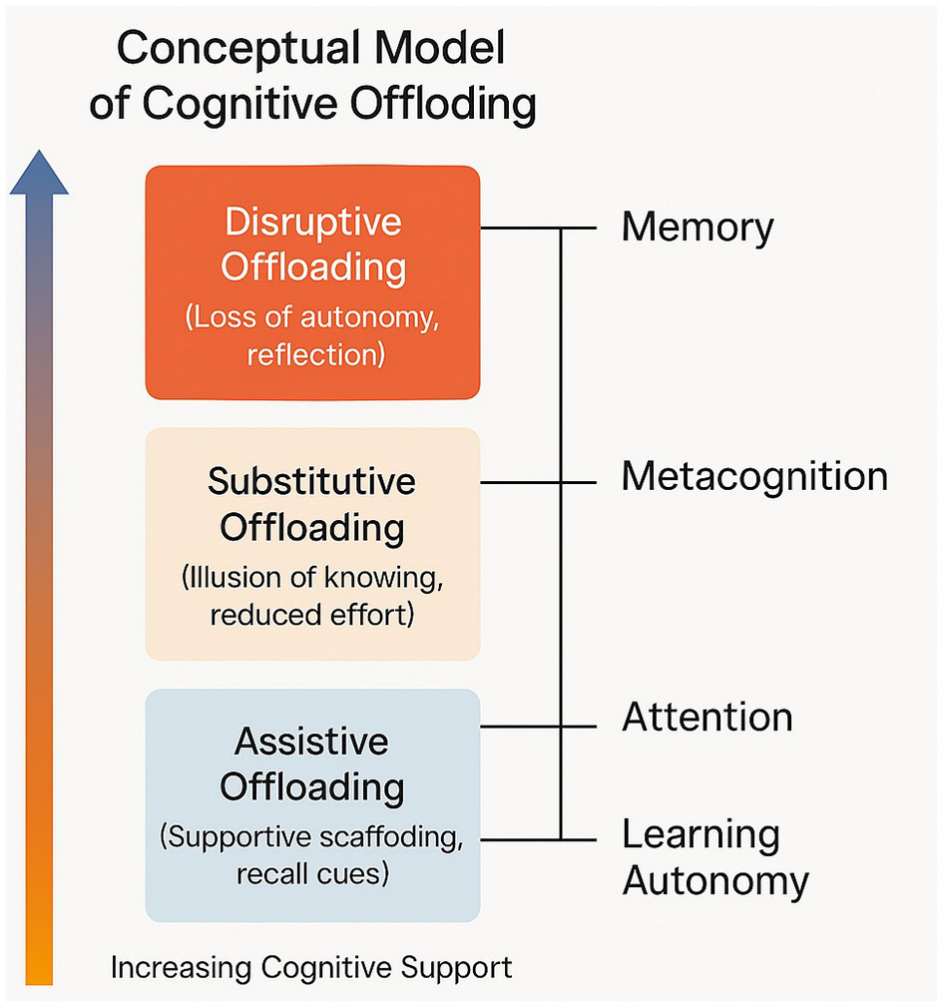
A visual overview of the proposed taxonomy.

A visual overview of the proposed taxonomy is presented in Figure 1.

The figure illustrates the continuum of cognitive offloading from assistive to disruptive forms, showing how increasing external automation progressively reduces internal control. Each level interacts with four core cognitive domains—memory, metacognition, attention, and learning autonomy—indicating that as offloading becomes more substitutive or disruptive, dependence on internal processes declines.

## Design ethics and cognitive sustainability

Designers will have to consider both what current AI systems enable as well as what they might replace when AI becomes integrated into our day-to-day decision making. As it relates to psychology, one of the primary concerns has been the potential for AI based decision support tools to gradually reduce the amount of effortful thinking required of users through their very nature as being low-friction and fast to produce results. Cognitive Load Theory (Sweller, 1988), for example, stresses the importance of achieving a cognitive “balance” of mental effort in order to optimize learning. Self-Determination Theory (Deci and Ryan, 2013), on the other hand, emphasizes that users require both competence and autonomy in order to develop motivationally and psychologically. If AI-based design consistently thwart this “cognitive balance,” then the likelihood increases that AI-based design could create an environment that encourages efficiency at the expense of personal growth and development.

In order to promote cognitive sustainability, AI-based interfaces need to incorporate “constructive friction” (Estaphan et al., 2025)—features that momentarily halt user interactions and prompt users to reflect upon their thoughts and/or recall past experiences. For example, AI-based interfaces could utilize delayed automatic suggested responses, confidence rating mechanisms, or brief prompts for minimal reflection before presenting AI-generated content. While these types of interface features may assist in maintaining cognitive engagement with users, they do so without hindering accessibility. Therefore, future research needs to assess how these seemingly minor design decisions affect cognitive processes in educational and health care environments, where internal agency is most important.

## Reinforcing cognitive agency through psychological training

In order to diminish reliance on AI, all future psychological interventions will focus on utilizing internal cognitive approaches that support a variety of cognitive processes. The following methods are examples of cognitive interventions that provide evidence of cognitive control; metacognitive training (Hertzog and Dunlosky, 2011), retrieval based learning and productive struggle. All digital literacy efforts should focus on providing more than technical ability. Efforts to develop “AI aware cognition”—the ability to identify when to use AI and when to continue with an effort—will foster learning resilience in an increasingly AI-enriched environment.

## Conclusion

While Cognitive Offloading is an intuitive and adaptable feature of human intelligence, it now represents a significant escalation in terms of frequency, scale, and psychological impact as a result of AI tools. The proposed conceptual framework identifies three forms of cognitive offloading (assistive, substitutive, and disruptive) to help clarify the ways in which delegating tasks can either support, replace, or diminish cognitive integrity. While the nature of the relationship between offloading and cognitive integrity is not directly relevant to the type of offloading occurring (i.e., assistive, substitutive, etc.), the degree to which individuals are aware of their actions and the extent to which they engage in such behavior are both crucial factors.

As we move through the four domains of memory, metacognition, attention and learning autonomy, the potential risks associated with offloading without purposeful consideration of the consequences become increasingly apparent. The cognitive tools that replace rather than enhance internal cognitive effort, also modify user's conceptions of task difficulty and distort perceptions of competence. Although adolescence presents a prime example of the potential for early delegation to impede the development of self-regulation and independent thought, these characteristics are not unique to adolescents; they represent a broad human tendency toward convenient cognition.

Psychologically speaking, these findings suggest a transition from a focus on the benefits of intelligent systems to the need for a new paradigm of cognitive sustainability; i.e., designing technologies and learning environments that preserve the effortful aspects of mental processing, promote reflection, and foster agency. Intelligent systems must therefore be judged not solely based upon their performance, but based on their impact on the quality of human thought surrounding that performance (Norman, 2024). There are many ways to encourage users to pause and reflect, including using interface features such as delayed suggestions, confidence checks, or reflection prompts, as well as integrating metacognitive training and retrieval-based learning into educational practices, to improve users' abilities to differentiate between times when to delegate and times when to continue the effortful mental work.

Ultimately, the future of human cognition in a world saturated with artificial intelligence will depend on the integration of both technological innovation and psychologically informed design. As we increasingly rely on machines to handle more of our mental lives, the fundamental question becomes: How much of our thinking should remain our own? In practical terms, this framework highlights the need for digital-literacy education that strengthens reflection rather than passive reliance. Educational policy should aim for cognitive sustainability, ensuring that AI systems are designed to complement, not replace, human learning and self-regulation.
